# Hypoxia-induced lncRNA MRVI1-AS1 accelerates hepatocellular carcinoma progression by recruiting RNA-binding protein CELF2 to stabilize SKA1 mRNA

**DOI:** 10.1186/s12957-023-02993-z

**Published:** 2023-03-28

**Authors:** Hang Tuo, Runkun Liu, Yufeng Wang, Wei Yang, Qingguang Liu

**Affiliations:** Department of Hepatobiliary Surgery, the First Affiliated Hospital of Xi’an Jiaotong University, Xi’an 710061, People’s Republic of China

**Keywords:** Hepatocellular carcinoma, MRVI1-AS1, SKA1, mRNA stability, Hypoxia

## Abstract

**Background:**

Long non-coding RNAs (lncRNAs) perform a vital role during the progression of hepatocellular carcinoma (HCC). Here, we aimed to identify a novel lncRNA involved in HCC development and elucidate the underlying molecular mechanism.

**Methods:**

The RT-qPCR and TCGA dataset analysis were applied to explore the expressions of MRVI1-AS1 in HCC tissues and cell lines. Statistical analysis was applied to analyze the clinical significance of MRVI1-AS1 in HCC. The functions of MRVI1-AS1 in HCC cells metastasis and growth were explored by transwell assays, wound healing assay, MTT assay, EdU assay, the intravenous transplantation tumor model, and the subcutaneous xenograft tumor model. Microarray mRNA expression analysis, dual luciferase assays, and actinomycin D treatment were used to explore the downstream target of MRVI1-AS1 in HCC cells. RIP assay was applied to assess the direct interactions between CELF2 and MRVI1-AS1 or SKA1 mRNA. Rescue experiments were employed to validate the functional effects of MRVI1-AS1, CELF2, and SKA1 on HCC cells.

**Results:**

MRVI1-AS1 was found to be dramatically upregulated in HCC and the expression was strongly linked to tumor size, venous infiltration, TNM stage, as well as HCC patients’ outcome. Cytological experiments and animal experiments showed that MRVI1-AS1 promoted HCC cells metastasis and growth. Furthermore, SKA1 was identified as the downstream targeted mRNA of MRVI1-AS1 in HCC cells, and MRVI1-AS1 increased SKA1 expression by recruiting CELF2 protein to stabilize SKA1 mRNA. In addition, we found that MRVI1-AS1 expression was stimulated by hypoxia through a HIF-1-dependent manner, which meant that MRVI1-AS was a direct downstream target gene of HIF-1 in HCC.

**Conclusion:**

In a word, our findings elucidated that hypoxia-induced MRVI1-AS1 promotes metastasis and growth of HCC cells via recruiting CELF2 protein to stabilize SKA1 mRNA, pointing to MRVI1-AS1 as a promising clinical application target for HCC therapy.

**Supplementary Information:**

The online version contains supplementary material available at 10.1186/s12957-023-02993-z.

## Introduction

Hepatocellular carcinoma (HCC) notoriously leads to more and more people’s death each year [1, 2]. The pathogenic factors are various, mainly including hepatitis B virus and excessive alcohol [2, 3]. However, the precise molecular mechanisms of HCC are not fully uncovered [4]. In consequence, it is pressing for us to figure out the pathogenesis of HCC.

Though long non-coding RNAs (lncRNAs) do not have the ability to encode proteins, it seems that we should not overlook their critical roles in living cells activities [5]. An increasing body of evidences suggest that lncRNAs involve in diverse processes of HCC cells [5–11]. In our previous findings, lncRNA MCM3AP-AS1, DSCR8, RUNX1-IT1, and CASC2 have been identified to be involved in HCC progression [12–15]. LncRNAs regulate gene expression through diverse molecular mechanisms at transcriptional or post-transcriptional level [5, 16–18]. It has been reported that under the mediation of RNA-binding proteins, lncRNAs could modulate the target mRNA stability [19–21]. For instance, lncRNA DANCR binds to RNA-binding protein 3 (RBM3) to stabilize SOX2 mRNA, then regulating cell proliferation in nasopharyngeal carcinoma [22]. LncRNA TSLNC8 promotes the binding of RNA-binding protein HuR with CTNNB1 mRNA and increased the stability of CTNNB1 mRNA, thus activating WNT/β-catenin signaling pathway in pancreatic cancer [23]. LncRNA PITPNA-AS1 promotes lung squamous cell carcinoma progression by recruiting TAF15 to stabilize HMGB3 mRNA [24]. Notably, based on our RNA-seq analysis data, lncRNA MRVI1-AS1 was identified as an oncogene in HCC, which has been reported to be associated with nasopharyngeal cancer chemoresistance [25]. MRVI1-AS1 inhibits miR-513a-5p miR-27b-3p to upregulate activating transcription factor 3 (ATF3), then increasing nasopharyngeal cancer’s sensitivity to paclitaxel by modulating the Hippo-TAZ signaling pathway [25]. However, the exact expression and functions of MRVI1-AS1 in HCC remain to be elaborated.

To conclude, this study identified a new lncRNA highly expressed in HCC, termed MRVI1-AS1. MRVI1-AS1 expression is not only closely related to the malignant clinicopathological features and outcomes of HCC but also a key promoter of HCC growth and metastasis. Furthermore, MRVI1-AS1 specifically recruits RNA-binding protein CELF2 to stabilize SKA1 mRNA, and MRVI1-AS1 is a HIF-1 target gene in HCC. Thus, our findings represent a novel therapeutic target strategy for HCC therapy.

## Materials and methods

### Tissue specimens

HCC tissue samples and adjacent non-tumor tissue samples, which were histopathologically confirmed, were collected from 72 patients who underwent surgery in the First Affiliated Hospital of Xi’an Jiaotong University from Jan. 2012 to Jan. 2014. All of the patients did not receive chemotherapy or radiotherapy before surgery. All of the samples were stored at −80℃. Our study got approval from the Ethics Committees of the First Affiliated Hospital of Xi’an Jiaotong University and written informed consent was obtained from all patients. The clinical parameters of HCC patients were shown in Table 1.

**Table 1:** Correlation between MRVI1-AS1 expression and the clinicopathologic characteristics of hepatocellular carcinoma

Characteristics		*n* = 72	MRVI1-AS1 expression		*P*
			Low (*n* = 36)	High (*n* = 36)	
Age (year)	<50	23	10	13	0.448
	≥50	49	26	23	
Gender	Male	61	30	31	0.743
	Female	11	6	5	
HBV infection	Absent	13	8	5	0.358
	Present	59	28	31	
Serum AFP level (ng/mL)	<20	17	11	6	0.165
	≥20	55	25	30	
Tumor size (cm)	<5	33	21	12	0.033*
	≥5	39	15	24	
Number of tumor nodules	1	60	33	27	0.058
	≥2	12	3	9	
Cirrhosis	Absent	19	12	7	0.181
	Present	53	24	29	
Venous infiltration	Absent	52	30	22	0.035*
	Present	20	6	14	
Edmondson–Steiner grading	I + II	47	26	21	0.216
	III + IV	25	10	15	
TNM stage	I + II	54	32	22	0.007*
	III + IV	18	4	14	

*HBV* Hepatitis B virus, *AFP* Alpha-fetoprotein, *TNM* Tumor-node-metastasis

^*^P < 0.05

### Cell culture

The human normal liver cell line (LO2) and five HCC cell lines (Hep3B, Huh7, SK-HEP-1, HepG2, and MHCC-97H) were obtained from the Cell Bank of the Chinese Academy of Sciences (Shanghai, China). All of the cells were maintained in an incubator (37℃, 5% CO_2_) and cultured in DMEM (Gibco, Grand Island, NY, USA) supplemented with 10% FBS (Gibco, Grand Island, NY, USA) and 1% penicillin-streptomycin (Invitrogen, CA, USA). All cell lines that we used in this study were tested and authenticated by DNA sequencing using the AmpF/STR method (Applied Biosystems) and tested for the absence of mycoplasma contamination (MycoAlert) and the latest date tested is 30 October 2022.

### Cell transfection

The full-length cDNA of MRVI1-AS1 was cloned into the pcDNA3.1 vector (GenePharma, Shanghai, China) to construct the MRVI1-AS1-overexpressed plasmid, and shRNA that specifically targeted HIF-1α, MRVI1-AS1, or SKA1 was cloned into the pLKO.1 vector (GenePharma). For lentiviral vector transduction, cells were seeded onto plate wells and infected with a l entiviral construct containing different vectors supplemented with 5 mg/ml polybrene (Gene-Pharma Co., Suzhou, China). Then the cells were selected with 5 mg/ml puromycin to create stable cell subclones, and all of the experimental operations were based on the product specifications.

### RT-qPCR

TRIzol reagent (Invitrogen, Carlsbad, CA) was used to isolate total RNA from tissue samples and cell lines based on the product manual. Then cDNA was obtained after reverse transcription. RT-qPCR was performed with SYBR Green Master Mixture (Takara, Dalian, China). GAPDH was used as the control. Relative gene expression levels were calculated using the 2^−ΔΔCt^ method. Primers for MRVI1-AS1: Forward: 5’-GCCCTGGTATTCCTTGAACA-3’, Reverse: 5’-TCAGTCCAGGAAGAGGT-3’. Primers for SKA1: Forward: 5’-CCTGAACCCGTAAAGAAGCCT-3’, Reverse: 5’-TCATGTACGAAGGAACACCATTG-3’. Primers for GAPDH: Forward: 5’-GGAGCGAGATCCCTCCAAAAT-3’, Reverse: 5’-GGCTGTTGTCATACTTCTCATGG-3’.

### Transwell assays

After being transfected with plasmids for 48 h, the cells were seeded into transwell chambers (8 µm pore size, Corning, USA) containing 200 µl medium with 1% FBS. The lower chambers were added with 800 µl medium containing 10% FBS. For detection of invasion ability, transwell chambers were pre-coated with Matrigel. Twenty-four hours later, cells passed through the membrane were stained with crystal violet (0.1%) and counted.

### Wound healing assay

Transfected cells were seeded into 6-well plates to form cell monolayers. When cell confluency reached to 80%, a 200-µl tip was used to scratch the cell layers. After being gently washed, cells were cultured with serum-free medium for 24 h. A microscope (IX71, Olympus, Tokyo, Japan) was used to image (magnification: 200×) the wounded gaps at 0 and 24 h after being created.

### Cell proliferation assay

For MTT assay, transfected cells were plated into 96-well plates (2000 cells/well). Then at 0, 24, 48, and 72 h after seeding, MTT (10 µL/well, Sigma, USA) was added to each well and incubated for 4 h at 37℃. Then, DMSO (100 µL/well, Sigma, USA) was used to dissolve the crystals. Absorbance was measured at 490 nm by a microplate reader (Bio-Rad, Richmond, CA). For EdU assay, Cell-Light™ EdU Apollo®567 In Vitro Imaging Kit (RiboBio Co., Ltd., Guangzhou, China) was used. Briefly, transfected HCC cells (1 × 10⁠^5^) were cultured in 96-well plates. Cells were incubated with EdU labeling medium at a moderate concentration for 2 h. Then, the cells were fixed with 4% paraformaldehyde, glycine, and 0.5% TritonX-100 in PBS. Next, cells were stained with 100 µL Apollo dye solution for 30 min at room temperature. The cells were subsequently stained using Hoechst and incubated for 30 min. The photos were taken on a microscope. The percentage of EdU-positive cells was calculated using ImageJ software.

### Luciferase reporter assay

To detect the effects of MRVI1-AS1 on luciferase activity of SKA1 promoter, full-length SKA1 promoter was cloned into pGL3 plasmid (pGL3-SKA1). pGL3 or pGL3-SKA1 with pRL-TK was transfected into MRVI1-AS1 overexpressing or MRVI1-AS1 knockdown HCC cells. After 48 h, the luciferase activities were measured using a dual-luciferase reporter gene assay system (Promega). The relative ratio of firefly luciferase activity to Renilla luciferase activity was measured.

### Subcellular localization of MRVI1-AS1

The separation of nuclear and cytosolic fractions was performed using the PARIS Kit (Life Technologies, Carlsbad, CA) according to the manufacturer’s instructions. Then, the subcellular localization of MRVI1-AS1 was detected by RT-qPCR. The GAPDH and U6 transcripts were used as an internal reference of cytoplasmic and nuclear RNA, respectively.

### RNA pull-down assay

RNA pull-down assay was performed using RNA-Protein Pull-Down Kit (Thermo Scientific) according to the manufacturer’s instructions. Briefly, biotin-labeled RNAs were in vitro transcribed, treated with RNase-free DNase I, and purified. Cell lysates were prepared using lysis buffer. Then, 1 mg cell lysates were mixed with 50 pmol of biotin-labeled RNAs. The washed streptavidin agarose beads were added to each binding reaction and further incubated at room temperature for 1 h. Beads were washed and boiled in sodium dodecyl sulfate buffer. The MRVI1-AS1-pull-down or antisense-MRVI1-AS1-pull-down protein samples were subjected to western blot with CELF2 antibody CELF2 (#NBP2-16035, Novus, USA). The antisense RNA of MRVI1-AS1 was taken as a negative control in RNA pull-down assay.

### RNA immunoprecipitation (RIP) assay

RIP assay was performed using the EZ-Magna RIP kit (Millipore, Billerica, MA) following the manufacturer’s protocol. HCC cells at 70–80% confluence were scraped off and then lysed in complete RIP lysis buffer. A total of 100 µl of whole cell extract was incubated with RIP buffer containing magnetic beads conjugated with antibodies against CELF2 (#ab156877, Abcam, USA) or control IgG (#ab172730, Abcam, USA) for 6 h at 4°C. The beads were then washed with washing buffer, and the complexes were incubated with 0.1% SDS/0.5 mg/ml Proteinase K (30 min at 55°C) to remove proteins. The immunoprecipitated RNAs were then extracted, and the RNA concentration and quality were determined by NanoDrop spectrophotometer (Thermo Scientific). Finally, immunoprecipitated RNA was analyzed by RT-qPCR.

### Western blot

Total proteins were isolated from cells with RIPA buffer (Beyotime, Hangzhou, China). Ten percent SDS-PAGE gels separated protein, then transferred to PVDF membranes (Millipore, Billerica, MA, USA). After being blocked by 5% nonfat milk for 2 h, antibodies for HIF-1α (1:1000, # ab228649, Abcam, USA), SKA1 (1:1000, #ab91550, Abcam, USA), CELF2 (1:1000, #NBP2-16035, Novus, USA),and β-actin (1:1000, # ab8226, Abcam, USA) were used to incubate membranes at room temperature overnight. Then, the membranes were incubated by the HRP-conjugated secondary antibodies. The blots were detected using an enhanced chemiluminescence reagent (Millipore, Billerica, MA, USA).

### Microarray mRNA expression analysis

Global mRNA expression was analyzed by the PrimeView Human Gene Expression Array (Affymetrix). Total RNA was converted into cRNA and labeled with biotin using MessageAmp Premier RNA Amplification Kit (#1792, Ambion) according to the manufacturer’s instructions. The fragmented cRNAs were hybridized on the gene chip, and then the chip was washed and stained following the manufacturer’s standard protocol. The fluorescent signal was scanned by GeneChip Scanner 3000 (Affymetrix) and converted into digital data (CEL) using Affymetrix GeneChip Command Console (AGCC) software. The resulting data were preprocessed using Robust Multi-array Average (RMA) algorithm. The fold change (FC) of gene expression in shMRVI1-AS1 cells was calculated relative to shNTC cells. A gene was defined as differentially expressed if its log2|FC| > 0.5.

### Chromatin immunoprecipitation assay (ChIP)

Hep3B and MHCC-97H cells were incubated at 20% or 1% O_2_ for 16 h, cross-linked in 3.7% formaldehyde for 15 min, quenched in 0.125 M glycine for 5 min, and lysed with SDS lysis buffer. Chromatin was sheared by sonication, and lysates were precleared with salmon sperm DNA/protein A agarose slurry (Millipore) for 1 h and incubated with antibody against HIF-1α (# ab228649, Abcam, USA) or IgG (#ab97051, Abcam, USA) in the presence of protein salt, high-salt, and LiCl buffers; DNA was 426 eluted in 1% SDS with 0.1 M NaHCO3, and cross-links were reversed by addition of 0.2 M NaCl. DNA was purified by phenol–chloroform extraction and ethanol 427 precipitation and analyzed by qPCR. Primers are as below: MRVI1-AS1-HRE-1-Forward: 5’-AGACGGGCGTCAATAGAATG-3’, MRVI1-AS1-HRE-1-Reverse: 5’-TTGCTAGCTGCTCCAGGACT-3’. MRVI1-AS1-HRE-2-Forward: 5’-TTAGCCGGGTCTCAAGGTAG-3’, MRVI1-AS1-HRE-2-Reverse: 5’-GGCTGGACACCCAAATAAGA-3’.

### Experiments in vivo

Nude mice (BALB/c, female, 4 weeks old) were adopted for the establishment of the intravenous transplantation tumor model and the subcutaneous xenograft tumor model. In the intravenous transplantation tumor model, the mice were inoculated with MHCC-97H subclones at a density of 2 × 10^5^ cells/100 µL through the tail vein. Five weeks after cell injection, the mice were euthanized, and the formation of metastatic lung nodes was observed and evaluated. In the subcutaneous xenograft tumor, MHCC-97H subclones cells (2 × 10^6^/200 µL) were subcutaneously injected into the right flank of mice. Then, the tumor growth was measured every week, and calipers were used to measure tumor length (*L*) and width (*W*), and tumor volume (*V*) was calculated as *V* = *L* × *W*^2^ × 0.524. Four weeks after cell injection, the mice were euthanized, then the tumor nodules were resected, and the tumor weight was measured. Part of the tumor nodule was stored at −80℃ for the detection of RT-qPCR, and the rest was fixed in 4% formaldehyde solution for immunohistochemical staining of Ki-67 (#ab238020, Abcam, USA). The protocols for the above mice experiments were approved by the Institutional Animal Ethical Committee of the Xi’an Jiaotong University.

### Statistical analysis

Graphpad Prism 8.0 (San Diago, CA, USA) and SPSS 20.0 (SPSS, Inc., Chicago, IL, USA) were applied to analyze the data. All of the data are presented as mean ± S.D. Statistical methods in this study included Student’s *t*-test, one-way ANOVA, Chi-square test, Kaplan–Meier method, log-rank test, and Pearson’s correlation coefficient analysis. The difference with* P *< 0.05 was considered to be statistically significant.

## Results

### Upregulated lncRNA MRVI1-AS1 indicates poor prognosis of HCC

**Fig. 1:**
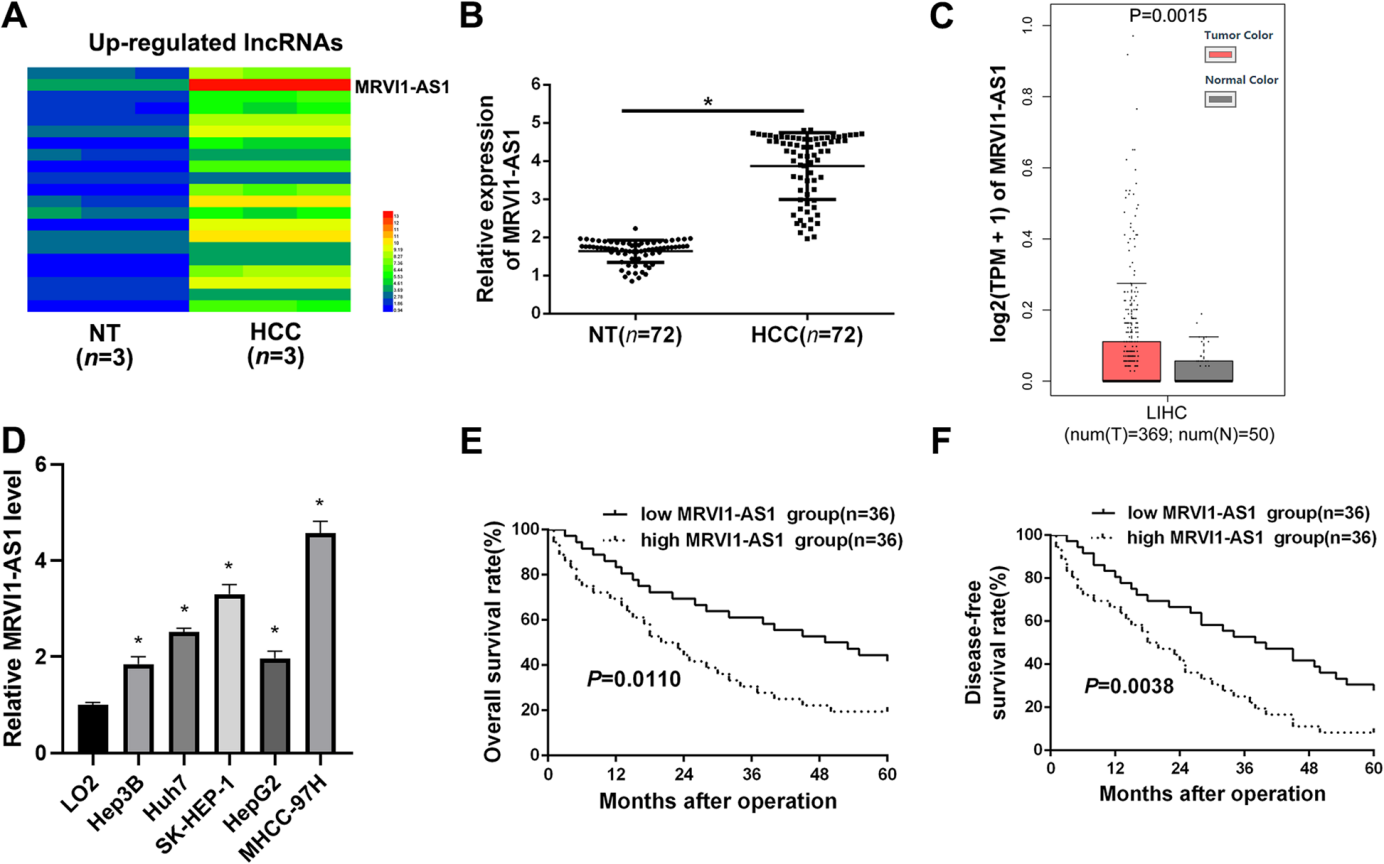
Upregulated lncRNA MRVI1-AS1 indicates poor prognosis of HCC. **A** RNA-seq analysis was used to identify the differentially expressed lncRNAs in HCC tissues (*n* = 3) and adjacent non-tumor (NT) tissues (*n* = 3), and the upregulated lncRNAs in HCC were shown as heat map. **B** RT-qPCR was conducted to explore the expression of MRVI1-AS1 in HCC tissues (*n* = 72) and adjacent non-tumor (NT) tissues (*n* = 72). *P < 0.05, Student’s *t* test. **C** TCGA data from GEPIA platform showed MRVI1-AS1 expression in HCC tissues (*n* = 369) and adjacent non-tumor tissues (*n* = 50). Student’s *t* test. **D** RT-qPCR was applied to explore the expression of MRVI1-AS1 in HCC cell lines (Hep3B, HuH7, SK-HEP-1, HepG2, and MHCC-97H) and normal hepatocyte cell line (LO2) (mean ± SD; *n* = 3). *P < 0.05, two-way ANOVA. **E**,** F** The effects of MRVI1-AS1 expression on 5-year overall survival and disease-free survival of HCC patients. Log-rank test

MicroArray or RNA-Seq was performed to analyze the abnormally expressed lncRNAs in HCC tissues and adjacent non-tumor (NT) tissues. The result indicated that MRVI1-AS1 was the one with the highest fold change increase among the upregulated lncRNAs in HCC (Fig. 1A). Furthermore, the data form RT-qPCR suggested MRVI1-AS1 was dramatically upregulated in HCC tissues, compared to that in non-tumor tissues (Fig. 1)B. Additionally, the analysis of TCGA data from GEPIA platform consistently found the higher expression of MRVI1-AS1 in HCC (Fig. 1C), and RT-qPCR results in HCC cell lines revealed that MRVI1-AS1 expressions in all of the five HCC cell lines (Hep3B, Huh7, SK-HEP-1, HepG2, and MHCC-97H) were dramatically higher than that in the human normal liver cell line (LO2) (Fig. 1D).

Then, we explored the correlation between the clinical significance and MRVI1-AS1 expression in HCC by sorting the 72 patients into low and high MRVI1-AS1 group on the basis of the median expression of MRVI1-AS1 in HCC tissues. Intriguingly, MRVI1-AS1 was closely related to tumor size, venous infiltration, and TNM stage (Table 1). Additionally, HCC patients with higher MRVI1-AS1 expression had both worse 5-year overall survival (OS) (Fig. 1E) and disease-free survival (DFS) (Fig. 1F). Thus, the above findings suggest that MRVI1-AS1 may promote HCC progression and development.

### MRVI1-AS1 promotes HCC metastasis and growth

**Fig. 2:**
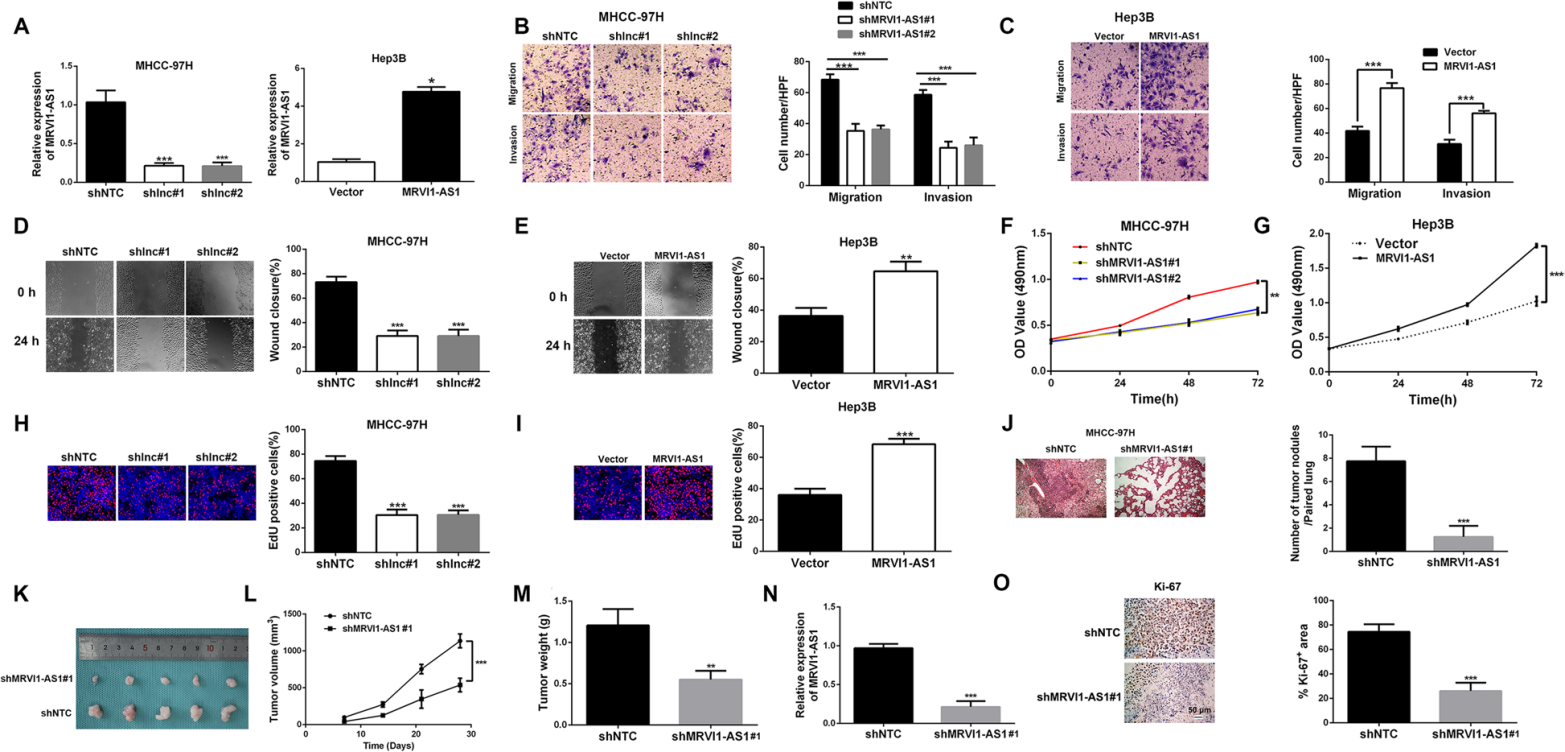
The effects of MRVI1-AS1 on metastasis and growth of HCC cells. **A** MRVI1-AS1 shRNAs (shMRVI1-AS1#1, shMRVI1-AS1#2) significantly decreased the expression of MRVI1-AS1 in MHCC-97H cells (mean ± SD; *n* = 3). ***P < 0.001, two-way ANOVA. The pcDNA/MRVI1-AS1 significantly increased the expression of MRVI1-AS1 in Hep3B cells (mean ± SD; *n* = 3). *P < 0.05, Student’s *t* test. **B**, **C** Transwell assays revealed that MRVI1-AS1 shRNAs notably suppressed migration and invasion of MHCC-97H cells (mean ± SD; *n* = 3). ***P<0.001, two-way ANOVA. MRVI1-AS1 overexpression notably promoted migration and invasion of Hep3B cells. (mean ± SD; *n* = 3). ***P < 0.001, Student’s *t* test. **D**, **E** Wound healing assays revealed that MRVI1-AS1 silencing markedly suppressed MHCC-97H cells mobility (mean ± SD; *n* = 3). ***P < 0.001, two-way ANOVA. Ectopic expression of MRVI1-AS1 obviously strengthened migration of Hep3B cells (mean ± SD; *n* = 3). **P < 0.01, Student’s *t* test. **F**, **G** MTT assays indicated that MRVI1-AS1 knockdown notably suppressed viability of MHCC-97H cells. Ectopic expression of MRVI1-AS1 markedly enhanced viability of Hep3B cells (mean ± SD; *n* = 3). **P<0.01, ***P<0.001, two-way ANOVA. **H**, **I** EdU assays revealed that MRVI1-AS1 silencing notably inhibited proliferation of MHCC-97H cells (mean ± SD; *n* = 3; ***P<0.001, two-way ANOVA), while MRVI1-AS1 overexpression had the contrary effect on Hep3B cells (mean ± SD; *n* = 3; ***P < 0.05, Student’s *t* test). **J** Nude mice were injected by MHCC-97H subclones through the tail vein. Then formation rate of tumor nodule in the lung was evaluated by lung section H&E staining (mean ± SD; *n* = 5). ***P < 0.001, Student’s *t* test. **K**, **L** MHCC‑97H subclones were injected subcutaneously into the right flank of nude mouse and tumor volume was measured every 7 days (mean ± SD; *n* = 5). ***P < 0.001, two-way ANOVA with Sidak’s *t* test. **M** Tumors were harvested on day 28 and weighed (mean ± SD; *n* = 5). **P < 0.01, two-way ANOVA. **N** Tumor RNA was isolated and RT-qPCR analysis of MRVI1-AS1 was performed. ***P < 0.001, Student’s *t* test. **O** Tumor sections were analyzed by immunohistochemistry for Ki-67, and the stained area in 10 fields was quantified by Image J software and the percentage of total area that was positive for staining is shown. ***P < 0.001, Student’s *t* test

Next, to further determine the role of MRVI1-AS1 in HCC, we firstly attempted to investigate whether MRVI1-AS1 was capable of promoting HCC cells invasion and migration. The MHCC-97H subclones stably expressing MRVI1-AS1 shRNAs or control, and Hep3B subclones stably expressing pcDNA/MRVI1-AS1 or control were produced by lentivirus transfection. Then, the knockdown and overexpression efficiencies were validated by RT-qPCR (Fig. 2A). Subsequently, transwell migration and invasion assays were performed. Results manifested that both migration and invasion abilities of MHCC-97H cells were repressed in MRVI1-AS1-knockdown subclones of MHCC-97H (Fig. 2B), while these two abilities were markedly enhanced by ectopic expression of MRVI1-AS1 in Hep3B cells (Fig. 2C). Consistently, the similar results were found in wound healing assay (Fig. 2D, E). Next, we attempted to explore the function of MRVI1-AS1 in HCC cells growth. MTT assay results manifested that MRVI1-AS1 shRNAs weakened MHCC-97H cells viability (Fig. 2F). In contrast, upregulated MRVI1-AS1 promoted Hep3B cells viability (Fig. 2G). Consistently, in EdU assay, the proportions of EdU-positive cells were much lower in MRVI1-AS1-knockdown subclones compared to the control group (Fig. 2H), while the proportion was much higher in MRVI1-AS1-overexpressing subclone (Fig. 2I). Furthermore, we established intravenous transplantation tumor model and subcutaneous xenograft tumor model to respectively examine whether MRVI1-AS1 promoted HCC cells metastasis and growth in vivo. The hematoxylin-eosin (H&E) staining results in lung tissues indicated that tumor nodules were less likely to form or grow bigger in lung tissue of MRVI1-AS1-knockdown mouse group (Fig. 2)J. Additionally, the tumor grew slower and the final tumor weight was obviously lighter in the mice with MRVI1-AS1 silencing compared to the control group (Fig. 2K-M). Expression of MRVI1-AS1 in NTC and MRVI1-AS1-knockdown tumors was validated by RT-qPCR (Fig. 2N). And HCC nodule tissues in MRVI1-AS1 knockdown group showed a weaker Ki-67 staining compared to NTC group (Fig. 2O). Collectively, these findings demonstrate that MRVI1-AS1 facilitates metastasis and growth of HCC cells.

### MRVI1-AS1 increases SKA1 expression through strengthening the stability of SKA1 mRNA

**Fig. 3:**
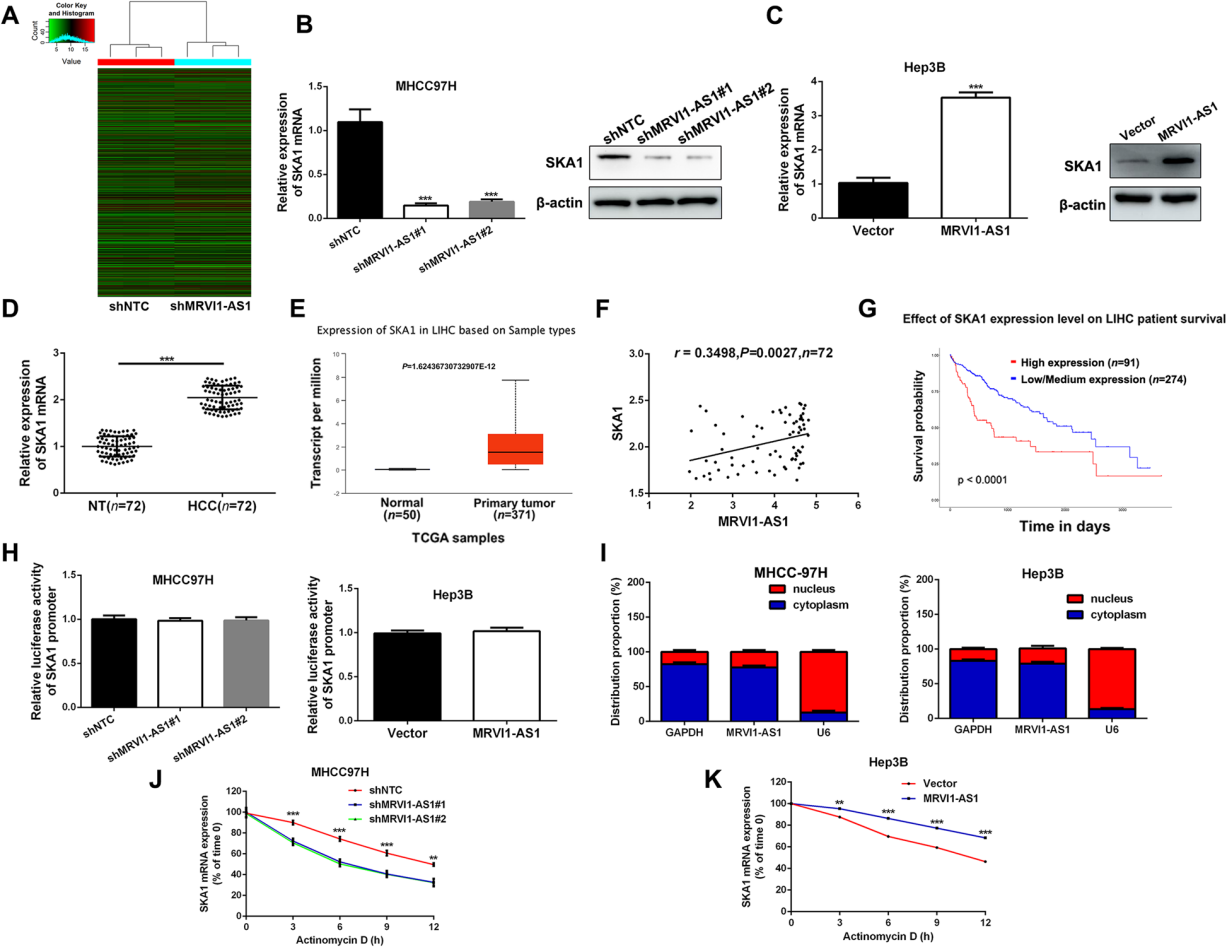
MRVI1-AS1 increases SKA1 expression by stabilizing SKA1 mRNA in HCC cells. **A** Heat map for downstream genes of MRVI1-AS1 in MHCC-97H cells. Among the downstream genes of MRVI1-AS1, the expression fold change of SKA1 is remarkable upon MRVI1-AS1 knockdown. **B**, **C** RT-qPCR and western blot were performed to detect mRNA and protein levels of SKA1 in MRVI1-AS1-knockdown subclones of MHCC-97H (mean ± SD; *n* = 3; ***P < 0.001, two-way ANOVA) and Hep3B cells (mean ± SD; *n* = 3; ***P < 0.001, Student’s *t* test). **D** RT-qPCR was conducted to explore the expression of SKA1 in HCC tissues (*n* = 72) and adjacent non-tumor (NT) tissues (*n* = 72). ***P < 0.001, Student’s *t* test. **E** TCGA data from UALCAN platform showed that MRVI1-AS1 expression in HCC tissues (*n* = 371) was significantly higher than that in normal tissues (*n* = 50). Student’s *t* test. **F** Pearson correlation analysis was applied to examine the correlation between MRVI1-AS1 and SKA1 mRNA in HCC tissues. **G** Data from UALCAN platform showed that HCC patients in high SKA1 expression group had worse prognosis. **H** Luciferase reporter assay was applied to detect whether MRVI1-AS1 influence the luciferase activity of SKA1 promoter (mean ± SD; *n* = 3). Student’s *t* test. **I** The separation of nuclear and cytosolic fractions assay was applied to determine the subcellular localization of MRVI1-AS1 in HCC cells (mean ± SD; *n* = 3). **J**, **K** HCC cells with MRVI1-AS1 alteration were treated with actinomycin D (Amyjet Scientific, Wuhan, China) to block RNA synthesis, and the degradation of SKA1 mRNA was examined using RT-qPCR assay at different time point (mean ± SD; *n* = 3). **P < 0.01, **P < 0.001, two-way ANOVA

Next, we sought to explo*r*e the potential mechanism of MRVI1-AS1 in HCC cells. The mRNA expression difference profile in MRVI1-AS1-knockdown MHCC-97H cells was examined by RNA-seq to identify the downstream targets. And SKA1 in particular caught our attention due to its remarkable expression fold change upon MRVI1-AS1 knockdown (Fig. 3A). Moreover, deletion of MRVI1-AS1 suppressed both mRNA and protein levels of SKA1 in MHCC-97H cells (Fig. 3B). In contrast, overexpression of MRVI1-AS1 increased both mRNA and protein levels of SKA1 in Hep3B cells (Fig. 3C). RT-qPCR analysis determined a higher expression of SKA1 in HCC tissues (Fig. 3D), and TCGA data analysis revealed the consistent result (Fig. 3E). In addition, in HCC tissues, MRVI1-AS1 expression was positively correlated with SKA1 expression (Fig. 3F), and data from UALCAN showed that high SKA1 expression had a close relationship with worse prognosis of HCC patients (Fig. 3G).

Subsequently, we tried to discover the underlying mechanism about how MRVI1-AS1 affected SKA1 expression. Firstly, we assumed that MRVI1-AS1 influenced SKA1 transcription in HCC cells, and the luciferase reporter containing SKA1 promoter was constructed. Unfortunately, the expression of MRVI1-AS1 had no influence on the luciferase activity of SKA1 promoter (Fig. 3H). Nevertheless, we determined that MRVI1-AS1 mainly localized in HCC cells cytoplasm (Fig. 3I), which suggested that MRVI1-AS1 might be associated with the stability of SKA1 mRNA. Furthermore, actinomycin D assay was performed in MRVI1-AS1-related HCC subclones, then isolated RNA was subjected to RT-qPCR analysis. As expected, the half-life of SKA1 mRNA was dramatically shortened in the situation of deletion of MRVI1-AS1 (Fig. 3J), while the half-life of SKA1 mRNA was prolonged by overexpressed MRVI1-AS1 (Fig. 3K). Collectively, these data suggest that MRVI1-AS1 increases SKA1 expression through strengthening the stability of SKA1 mRNA.

### MRVI1-AS1 recruits CELF2 protein to stabilize SKA1 mRNA

**Fig. 4:**
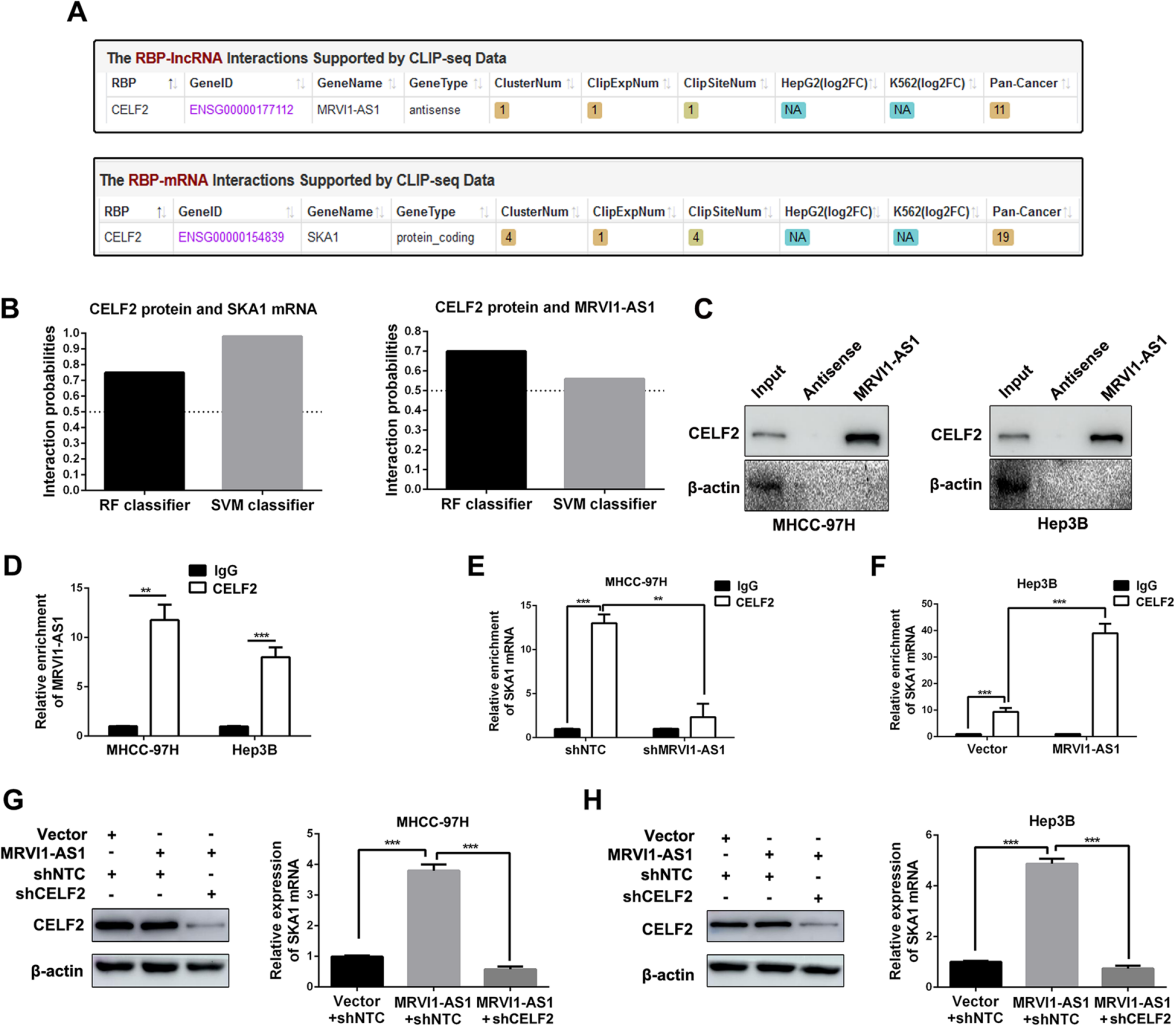
MRVI1-AS1 recruits CELF2 to stabilize SKA1 mRNA in HCC cells. **A** Data from the public datasets StarBase (http://starbase.sysu.edu.cn) showed that CELF2 was the potential RNA-binding protein for both MRVI1-AS1 and SKA1 mRNA. **B** Public platform RPIseq (http://pridb.gdcb.iastate.edu/RPISeq) was used to predict the interaction probability between CELF2 protein and MRVI1-AS1 or SKA1 mRNA (the interaction probabilities > 0.5 means positive result. *RF* random forest, *SVM* support vector machines). **C** RNA pull-down assay was performed by using biotin-labeled MRVI1-AS1 and the antisense-MRVI1-AS1 to assess the interaction between CELF2 protein and MRVI1-AS1. **D** RIP assay using antibody against CELF2 was used to explore the interaction between lncRNA MRVI1-AS1 and CELF2 protein in MHCC-97H and Hep3B cells. IgG served as the control. Then, immunoprecipitated RNA was purified and analyzed by RT-qPCR (mean ± SD; *n* = 3). **P<0.01, **P<0.001, Student’s *t* test. **D–F** RIP assay using antibody against CELF2 was used to explore the interaction between lncRNA MRVI1-AS1 and CELF2 protein in MRVI1-AS1 knockdown or MRVI1-AS1-overexpressing subclones of MHCC-97H and Hep3B cells. IgG served as the control. Then, immunoprecipitated RNA was purified and analyzed by RT-qPCR (mean ± SD; *n* = 3). **P<0.01, **P<0.001, two-way ANOVA. **G**, **H** Western blot and RT-qPCR analysis were applied to determine the regulatory relationships among MRVI1-AS1, CELF2, and SKA1 mRNA (mean ± SD; *n* = 3). **P < 0.001, **P < 0.001, two-way ANOVA

It has been reported that mRNA could be stabilized by RNA-binding proteins, which are recruited by lncRNAs [22–24]. Thus, we made the assumption that MRVI1-AS1 could enhance SKA1 mRNA stability mediated by a certain kind of RNA-binding protein. Firstly, StarBase (http://starbase.sysu.edu.cn) and RPISeq (http://pridb.gdcb.iastate.edu/RPISeq) were employed to predict the potential RNA-binding protein, which bond to both MRVI1-AS1 and SKA1 mRNA. The data showed that CELF2 was the potential RNA-binding protein for both MRVI1-AS1 and SKA1 mRNA (Fig. 4A, B). The binding of MRVI1-AS1 with CELF2 protein was verified by western blot analysis following the RNA pull-down assay both in MHCC-97H and Hep3B cells (Fig. 4C). Data of RIP assay indicated that both MRVI1-AS1 and SKA1 mRNA were enriched by the antibody against CELF2 (Fig. 4D-F). Furthermore, the interaction between CELF2 and SKA1 mRNA in MHCC-97H cells was impaired in the absence of MRVI1-AS1 (Fig. 4E), while the interaction was strengthened in Hep3B cells with overexpression of MRVI1-AS1 (Fig. 4F). In addition, western blot data revealed that SKA1 mRNA expression, but not CELF2, was increased by pcDNA/MRVI1-AS1, and the induction was abrogated by CELF2 shRNA (Fig. 4G, H). Taken together, we conclude that MRVI1-AS1 increases SKA1 expression by recruiting RNA-binding protein CELF2 to stabilize SKA1 mRNA.

### MRVI1-AS1 promotes HCC cells metastasis and growth under the mediation of SKA1

**Fig. 5:**
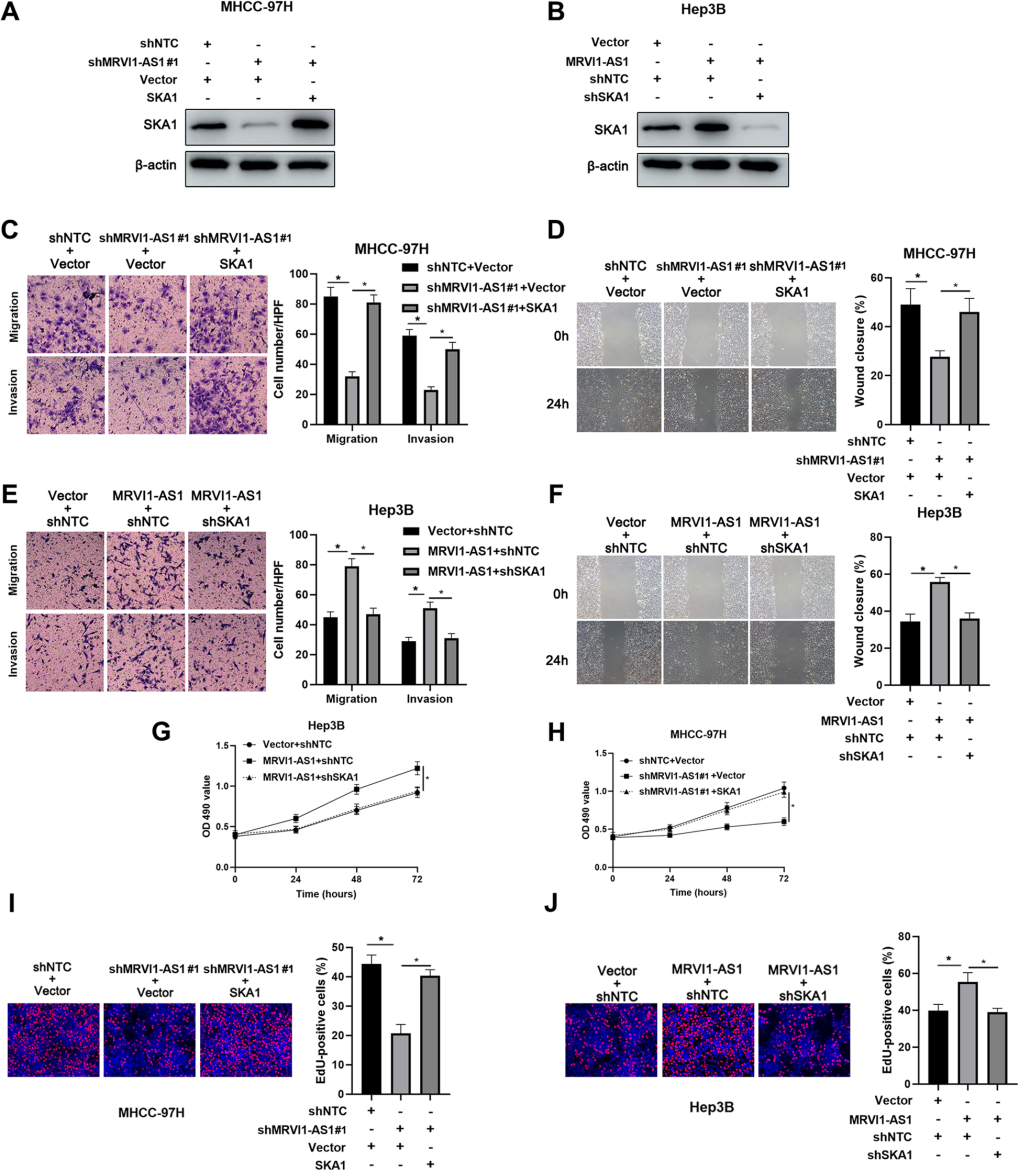
SKA1 mediates the effects of MRVI1-AS1 on HCC cells metastasis and growth. **A**, **B** SKA1 expression in MHCC-97H and Hep3B cells co-transfected with indicated vectors was detected by western blot. **C**, **E** Transwell assays revealed that SKA1 mediated the effects of MRVI1-AS1 on migration and invasion of HCC cells (mean ± SD; *n* = 3). *P < 0.05, two-way ANOVA. **D**, **F** Wound healing assays revealed that SKA1 mediated the effect of MRVI1-AS1 on migration of HCC cells (mean ± SD; *n* = 3). *P < 0.05, two-way ANOVA. **G**, **H** MTT assays revealed that SKA1 mediated the effect of MRVI1-AS1 on viability of HCC cells (mean ± SD; *n* = 3). *P < 0.05, two-way ANOVA. **I**, **J** EdU assays revealed that SKA1 mediated the effect of MRVI1-AS1 on proliferation of HCC cells (mean ± SD; *n* = 3). *P < 0.05, two-way ANOVA

Next, we attempted to validate that SKA1 mediated the influences of MRVI1-AS1 on HCC cells. As expected, MRVI1-AS1-knockdown inhibited SKA1 expression, and the repression was blocked by SKA1 overexpressing in MHCC-97H cells (Fig. 5A). On the other hand, SKA1 expression was increased by overexpressed MRVI1-AS1, and the induction was abrogated by SKA1 shRNA in Hep3B cells (Fig. 5B). Moreover, data from transwell assays and wound healing assay collectively manifested that SKA1 overexpression significantly reversed the suppression of MHCC-97H cells migration and invasion by MRVI1-AS1 silencing (Fig. 5C, D). SKA1 silencing offset the positive effects of MRVI1-AS1 overexpression on the migrated ability and invasive ability of Hep3B cells (Fig. 5E, F). In addition, the promoting effect of pcDNA/MRVI1-AS1 on Hep3B cells viability was abrogated by SKA1 silencing, as confirmed by MTT assay (Fig. 5G). In contrast, MRVI1-AS1 shRNA inhibited MHCC-97H cells viability, but SKA1 overexpression reversed the inhibitory effects on (Fig. 5H). Similarly, in EdU assay, the inhibitory effect of MRVI1-AS1 shRNA on MHCC-97H cell proliferation was determined, which then was rescued by SKA1 overexpression (Fig. 5I). SKA1 silencing offset the promoting effects of pcDNA/MRVI1-AS1 on Hep3B cell viability (Fig.5J). In brief, these findings suggest that MRVI1-AS1 promotes HCC cells metastasis and growth under the mediation of SKA1.

### Hypoxia induces MRVI1-AS1 expression through HIF-1-depedent manner in HCC cells

**Fig. 6:**
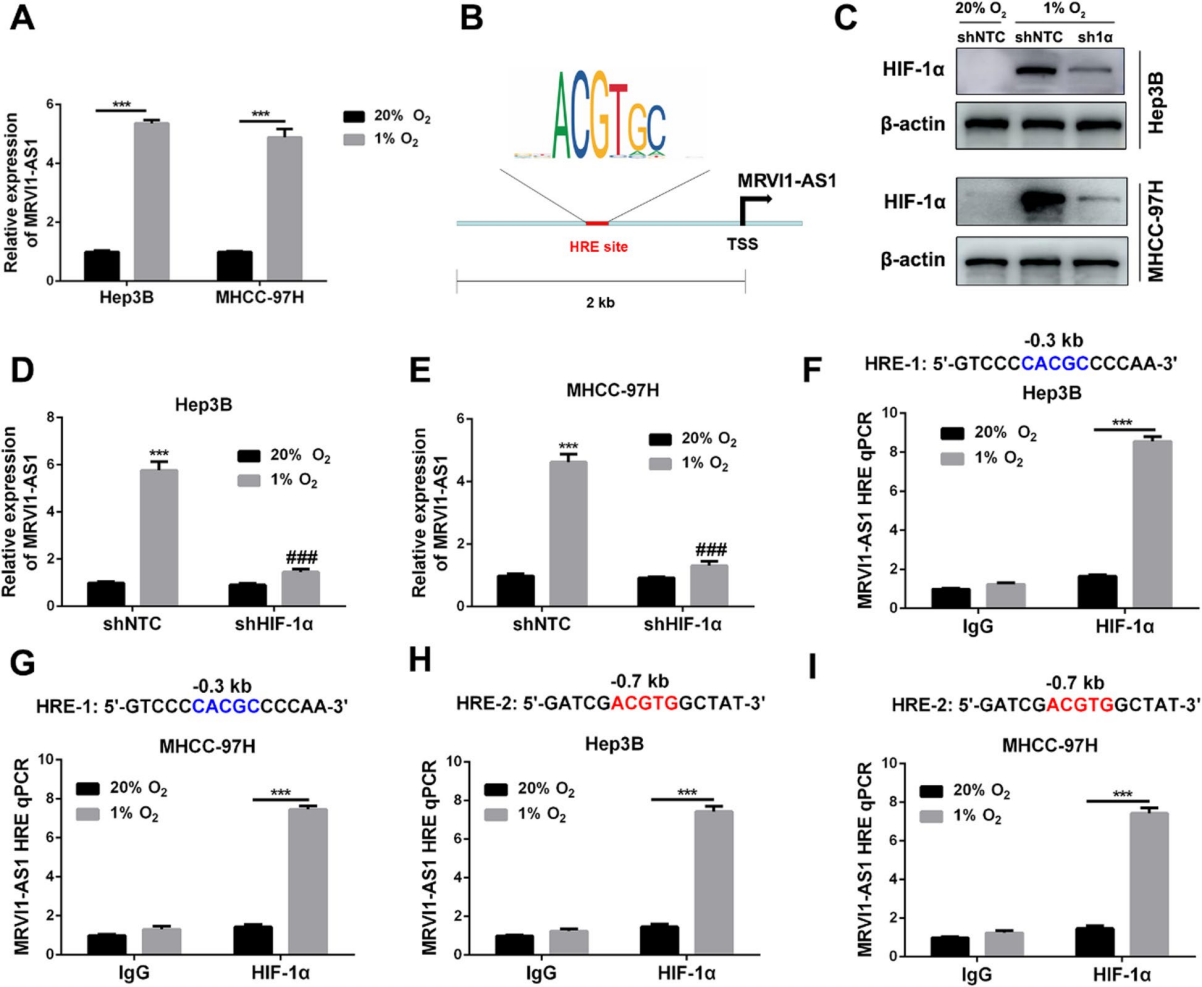
MRVI1-AS1 is induced by hypoxia in a HIF-1-depedent manner in HCC cells. **A** Hep3B and MHCC-97H cells were exposed to 20% or 1% O_2_ for 24 h, followed by RT-qPCR (mean ± SD; *n* = 3). ***P < 0.001, Student’s *t* test. **B** JASPAR database was applied to analyze whether there existed potential hypoxia-response element (HRE) in the promoter region of MRVI1-AS1 gene. **C** Hep3B and MHCC-97H subclones expressing a non-targeting control (NTC) shRNA or a shRNA targeting HIF-1a (sh1a) were exposed to 20% or 1% O_2_ for 8 h and western blot was performed. **D**, **E** Hep3B and MHCC-97H subclones were exposed to 20% or 1% O_2_ for 24 h, followed by RT-qPCR analysis for MRVI1-AS1 (mean ± SD; *n* = 3). ***P < 0.001 vs. NTC at 20% O_2_; ^###^*P* < 0.001 vs. NTC at 1% O_2_ (two-way ANOVA). **F–I** Hep3B and MHCC-97H cells were exposed to 20% or 1% O_2_ for 16 h, and ChIP assays were performed by using antibody against HIF-1α or IgG. Primers encompassing HIF binding sites located 0.3 kb 5’, 0.7 kb 5’ to the MRVI1-AS1 transcription start site (TSS) were used for qPCR. Results were normalized to the 848 first lane (mean ± SD; *n* = 3). ***P < 0.001, two-way ANOVA

In cancer, as a transcriptional trigger for numerous genes, including lncRNAs, hypoxic microenvironment addresses more and more attention to researchers [26, 27]. Here, we attempted to explore whether MRVI1-AS1 could be regulated by hypoxia. We exposed Hep3B and MHCC-97H cells to normoxia (20% O_2_) or hypoxia (1% O_2_) for 24 h and isolated RNA from these cells. RT-qPCR analysis data indicated that MRVI1-AS1 expressions in both Hep3B and MHCC-97H cells were significantly facilitated by hypoxia (Fig. 6A). Then, in order to determine whether HIF-1α mediated the induction of MRVI1-AS1 by hypoxia, the candidate hypoxia-response element (HRE) in the promoter region of MRVI1-AS1 gene was discovered by JASPAR database. Data indicated that 9 candidate HRE sites were predicted in the promoter region of MRVI1-AS1 gene (Fig. 6B, Supplemental Figure (1)), suggesting that MRVI1-AS1 could be a HIF-1 target gene. After the construction of HIF-1α-knockdown subclones, western blot was employed to verify the knockdown efficiency (Fig. 6C). In shNTC subclones of Hep3B and MHCC-97H cells, hypoxia increased MRVI1-AS1 levels, and the inductions were counteracted by HIF-1α silencing (Fig. 6D, E). Furthermore, ChIP assays were conducted in Hep3B and MHCC-97H cells, which had been exposed to 20% or 1% O_2_ for 16 h. The two HRE sites located 0.3 kb 5’ and 0.7 kb 5’ to the transcription start site (TSS) were identified (Fig. 6F-I), suggesting that HIF-1α and HRE sites were essential for MRVI1-AS1 transcription under hypoxia. Taken together, we demonstrate that hypoxia induces MRVI1-AS1 expression through HIF-1-depedent manner in HCC cells.

### Hypoxia promotes HCC progression through MRVI1-AS1/SKA1 pathway

**Fig. 7:**
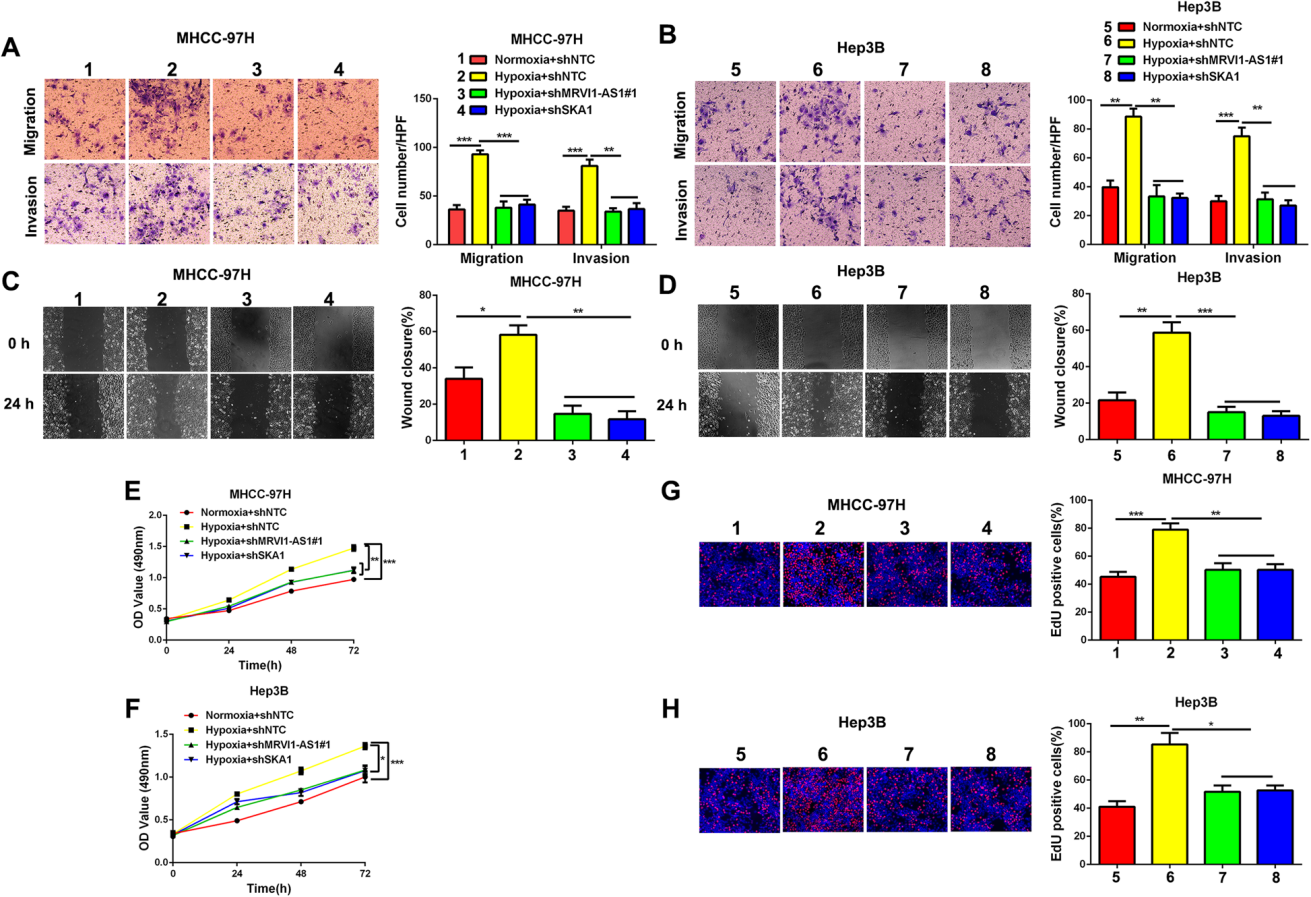
Hypoxia promotes HCC progression through MRVI1-AS1/SKA1 pathway. **A**, **B** Transwell assays were performed by using the indicated MHCC-97H and Hep3B subclones which were incubated in 20% O_2_ or 1% O_2_ for 24 h (mean ± SD; *n* = 3). **P < 0.01, ***P < 0.001, two-way ANOVA. **C**, **D** Wound healing assays were performed by using the indicated MHCC-97H and Hep3B subclones which were incubated in 20% O_2_ or 1% O_2_ for 24 h (mean ± SD; *n* = 3). *P < 0.05, **P < 0.01, ***P < 0.001, two-way ANOVA. **E**, **F** MTT assays were performed by using the indicated MHCC-97H and Hep3B subclones which were incubated in 20% O_2_ or 1% O_2_ (mean ± SD; *n* = 3). *P < 0.05, **P < 0.01, ***P < 0.001, two-way ANOVA. **G**, **H** EdU assays were performed by using the indicated MHCC-97H and Hep3B subclones which were incubated in 20% O_2_ or 1% O_2_ for 24 h (mean ± SD; *n* = 3). *P < 0.05, **P < 0.01, ***P < 0.001, two-way ANOVA

In order to investigate whether MRVI1-AS1/SKA1 pathway mediated HCC progression induced by hypoxia, we conducted a series of rescue experiments. Rescue experiments of transwell assays revealed that hypoxia dramatically promoted MHCC-97H and Hep3B cells migration and invasion, while MRVI1-AS1-knockdown or SKA1-knockdown counteracted the promoting effects of hypoxia on HCC cells migration and invasion (Fig. 7A, B). Consistently, wound healing assay indicated that MRVI1-AS1-knockdown or SKA1-knockdown offset the promoting effects of hypoxia on MHCC-97H and Hep3B cells mobility (Fig. 7C, D). In addition, MTT assay (Fig. 7E, F) and Edu assay (Fig.7G, H) indicated that MHCC-97H and Hep3B cells proliferation were accelerated by hypoxia, while MRVI1-AS1-knockdown or SKA1-knockdown abrogated the promoting effects. Thus, we demonstrated that hypoxia promotes HCC progression through MRVI1-AS1/SKA1 pathway.

## Discussion

The critical importance of lncRNAs in the process of HCC tumorigenesis has been elucidated by a large body of research evidence, which proposes a new hopefulness to HCC targeted therapy [5, 9, 28]. Though some lncRNAs related to HCC progression, such as CASC2, DSCR8, and MCM3AP-AS1, have been identified by our research team, further investigations are required. [12–14]. In this study, a novel lncRNA, termed MRVI1-AS1, was identified by our RNA-seq data analysis. The high expression was consistently verified both in a cohort of HCC tissues collected in the hospital and a cohort of HCC tissues from TCGA, as well as the HCC cell lines. MRVI1-AS1 has been reported to be associated with nasopharyngeal cancer sensitivity to paclitaxel by regulating the Hippo-TAZ signaling pathway [25], which suggests the close association of MRVI1-AS1 with tumor progression to some extent. Intriguingly, a few of clinical features, including tumor size, venous infiltration, and TNM stage, were found to be closely related to MRVI1-AS1 expression in HCC. Additionally, worse outcomes were presented in the HCC patients with higher MRVI1-AS1 expression. These findings collectively hinted the critical importance of MRVI1-AS1 in HCC development and the acceleration roles in HCC metastasis and growth, which were subsequently validated by a series of experiments in vitro and in vivo.

LncRNAs present its crucial importance through the multifaceted effects and various molecular mechanisms at transcriptional and post-transcriptional levels [28]. More and more studies reveal the existence of a widespread interaction network involving lncRNAs, where lncRNAs recruit binding proteins to stabilize the downstream target mRNA [9, 19]. For example, studies have presented the incremental stabilization of SOX2 mRNA induced by binding of lncRNA DANCR to RNA-binding protein 3 (RBM3), the increased stability of CTNNB1 mRNA mediated by the binding of lncRNA TSLNC8 to HuR, and the enhancive stability of HMGB3 mRNA induced by the binding of lncRNA PITPNA-AS1 to TAF15 [22–24]. In this study, microarray mRNA expression analysis identified SKA1 as a potential downstream target of MRVI1-AS1 in HCC. Subsequently, the overexpression of SKA1 in HCC was determined, and SKA1 mRNA expression was found to be positively related to MRVI1-AS1 expression in HCC. Furthermore, SKA1 expression was regulated by MRVI1-AS1 due to the mRNA stability modulation by MRVI1-AS1, but not the transcription activity.

RNA-binding proteins play critical roles in mRNA stability regulated by lncRNA [29, 30]. Here, StarBase (http://starbase.sysu.edu.cn) and RPISeq (http://pridb.gdcb.iastate.edu/RPISeq) were applied to uncover the latent RNA-binding protein, which bond to both MRVI1-AS1 and SKA1 mRNA. Data indicated that CELF2 might be the potential RNA-binding protein for MRVI1-AS1 and SKA1 mRNA, and it has been reported that CELF2 acts as the RNA-binding protein to mediate the regulation effect of GAS5 on VAV1 mRNA expression [31]. Here, we found that both MRVI1-AS1 and SKA1 mRNA were enriched by CELF2 protein, and the enrichment of SKA1 mRNA by CELF2 protein was abrogated by MRVI1-AS1 knockdown, while enhanced by MRVI1-AS1 overexpressing. In addition, MRVI1-AS1 had no effect on CELF2 expression. In brief, our data demonstrate that MRVI1-AS1 regulates SKA1 expression through recruiting RNA-binding protein CELF2 to affect the stability of SKA1 mRNA.

As a microtubule-binding protein of the outer kinetochore, SKA1 plays vital roles in the stabilization of kinetochore-spindle microtubule attachment, as well as proper chromosome segregation in the process of mitosis. In the previous studies, SKA1 has been identified as an oncogene in HCC [32, 33]. For example, Xiao J et al. found that SKA1 mediates the functions of LINC00339 and miR-1182 in HCC [34]. Here, through rescue experiments, we not only determined the oncogene role of SKA1 in HCC but also further affirmed the finding that SKA1 acted as the downstream target of MRVI1-AS1.

Intratumoral hypoxia powerfully stimulates the progression of HCC, during which hypoxia-inducible factors (HIFs) play a central role [35]. As a transcriptional regulatory factor, HIF-1 plays an important role in regulating the transcription of target genes, including lncRNAs [26, 27]. Here, we found that MRVI1-AS1 expression was increased by hypoxia, and hypoxia induced MRVI1-AS1 in a HIF-1-dependent manner. Furthermore, rescue experiments indicated that MRVI1-AS1-knockdown or SKA1-knockdown abrogated the promoting effects of hypoxia on HCC progression which meant hypoxia promoted HCC progression through MRVI1-AS1/SKA1 pathway. Thus, these findings suggest that hypoxia at least is one of the motivator for upregulation of MRVI1-AS1 in HCC.

## Conclusion

In HCC, hypoxia induced MRVI1-AS1 expression in a HIF-1-dependent manner, and overexpressed MRVI1-AS1 increased SKA1 expression by recruiting RNA-binding protein CELF2 to stabilize SKA1 mRNA, then promoting HCC progression. Our study has delineated a novel molecular mechanism and signaling pathway involved in HCC progression.

## Supplementary Information

**Additional file 1:** **Supplemental Figure 1.** MRVI1-AS1 is a HIF-1 target gene. Data from (https://jaspar.genereg.net) indicated that there existed 9 putative HRE sites in the promoter of MRVI1-AS1 gene for HIF-1 to bind to.

## Data Availability

The data used for supporting the findings of this study are available from the corresponding authors upon request.
