# Supplementary material for: Hypoxia-induced lncRNA MRVI1-AS1 accelerates hepatocellular carcinoma progression by recruiting RNA-binding protein CELF2 to stabilize SKA1 mRNA
Source: World J Surg Oncol. 2023 Mar 28;21:111. doi: 10.1186/s12957-023-02993-z (PMC10044719; doi:10.1186/s12957-023-02993-z)
Supplement: Supplementary file 1 — Additional file 1: Supplemental Figure 1. MRVI1-AS1 is a HIF-1 target gene. Data from (https://jaspar.genereg.net) indicated that there existed 9 putative HRE sites in the promoter of MRVI1-AS1 gene for HIF-1 to bind to. [file 12957_2023_2993_MOESM1_ESM.docx]

**
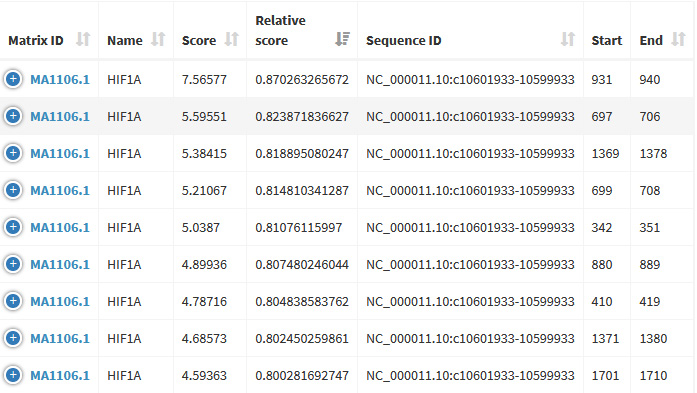
**

**Supplemental figure 1.** MRVI1-AS1 is a HIF-1 target gene. Data from (https://jaspar.genereg.net) indicated that there existed 9 putative HRE sites in the promoter of MRVI1-AS1 gene for HIF-1 to bind to.
